# Biologically Younger Individuals, as Identified by MARK‐AGE Biological Age Scores, Display a Distinct Favourable Blood Chemistry Profile Regardless of Age

**DOI:** 10.1111/acel.70437

**Published:** 2026-03-13

**Authors:** María Moreno‐Villanueva, Michael Junk, Grażyna Mosieniak, Ewa Sikora, Miriam Capri, Paolo Garagnani, Chiara Pirazzini, Nicolle Breusing, Jürgen Bernhardt, Christiane Schön, María Blasco, Gerben Zondag, Florence Debacq‐Chainiaux, Beatrix Grubeck‐Loebenstein, Birgit Weinberger, Simone Fiegl, Eugenio Mocchegiani, Marco Malavolta, Robertina Giacconi, Francesco Piacenza, Sebastiano Collino, Efstathios S. Gonos, Daniela Gradinaru, Martijn E. T. Dollé, Eugène Jansen, Michel Salmon, Peter Kristensen, Helen Griffiths, Claude Libert, Valerie Vanhooren, Andreas Simm, Duncan Talbot, Paola Caiafa, Maria Giulia Bacalini, Michele Zampieri, Bertrand Friguet, Isabelle Petropoulos, P. Eline Slagboom, Rudi Westendorp, Antti Hervonnen, Mikko Hurme, Richard Aspinall, Sheila Govind, Daniela Weber, Wolfgang Stuetz, Jan H. J. Hoeijmakers, Iuliia Gavriushina, Oliver R. Sampson, Gastone Castellani, Michael R. Berthold, Tilman Grune, Claudio Franceschi, Alexander Bürkle

**Affiliations:** ^1^ Molecular Toxicology Group, Department of Biology University of Konstanz Konstanz Germany; ^2^ Human Performance Research Centre, Department of Sport Science University of Konstanz Konstanz Germany; ^3^ Department of Mathematics and Statistics University of Konstanz Konstanz Germany; ^4^ Laboratory of the Molecular Bases of Ageing Nencki Institute of Experimental Biology, Polish Academy of Sciences Warsaw Poland; ^5^ Department of Medical and Surgical Sciences (DIMEC), Alma Mater Studiorum, University of Bologna Bologna Italy; ^6^ Alma Mater Research Institute on Global Challenges and Climate Change (Alma Climate), University of Bologna Bologna Italy; ^7^ Institute of Nutritional Medicine University of Hohenheim Stuttgart Germany; ^8^ BioTeSys GmbH Esslingen Germany; ^9^ Spanish National Cancer Research Centre (CNIO) Madrid Spain; ^10^ DNage BV (Extinct) Leiden the Netherlands; ^11^ University of Namur, Research Unit on Cellular Biology Namur Belgium; ^12^ Research Institute for Biomedical Aging Research Universität Innsbruck Innsbruck Austria; ^13^ UMIT TIROL—Private University for Health Sciences, Medical Informatics and Technology Hall in Tirol Austria; ^14^ Advanced Technology Center in Aging Research at IRCCS‐INRCA Ancona Italy; ^15^ Advanced Technology Center in Aging Research and Geriatric Mouse Clinic, IRCCS INRCA Ancona Italy; ^16^ Department of Clinical and Molecular Sciences, DISCLIMO Università Politecnica Delle Marche Ancona Italy; ^17^ Nestlé Research, Nestlé Institute of Health Sciences Lausanne Switzerland; ^18^ National Hellenic Research Foundation Institute of Biology, Medicinal Chemistry and Biotechnology Athens Greece; ^19^ Ana Aslan National Institute of Gerontology and Geriatrics, Bucharest Romania; Carol Davila University of Medicine and Pharmacy, Faculty of Pharmacy Bucharest Romania; ^20^ Centre for Health Protection, National Institute for Public Health and the Environment Bilthoven the Netherlands; ^21^ Straticell, Science Park Crealys Gembloux Belgium; ^22^ University of Aaarhus, and Department of Chemistry and Bioscience Aalborg University Aalborg Denmark; ^23^ Life and Health Sciences, Aston Research Centre for Healthy Ageing Aston University Birmingham UK; ^24^ Swansea University Swansea Wales UK; ^25^ VIB Center for Inflammation Research, Ghent, Belgium; and Department of Biomedical Molecular Biology Ghent University Ghent Belgium; ^26^ Department of Cardiothoracic Surgery University Hospital Halle Halle (Saale) Germany; ^27^ Unilever R&D, Colworth Science Park Sharnbrook UK; ^28^ Department of Cellular Biotechnologies and Haematology Sapienza University of Rome Rome Italy; ^29^ Department of Biomedical and NeuroMotor Sciences (DiBiNeM) University of Bologna Italy; ^30^ Department of Experimental Medicine Sapienza University of Rome Rome Italy; ^31^ Sorbonne Université, CNRS, Inserm, Biological Adaptation and Ageing—IBPS Paris France; ^32^ Section of Molecular Epidemiology Leiden University Medical Centre Leiden the Netherlands; ^33^ Department of Gerontology and Geriatrics Leiden University Medical Center Leiden the Netherlands; ^34^ Department of Public Health and Center for Healthy Aging University of Copenhagen Copenhagen Denmark; ^35^ Faculty of Medicine and Health Technology University of Tampere Tampere Finland; ^36^ Cranfield University, and Centre for Intelligent Healthcare, Coventry University Coventry UK; ^37^ Cranfield University, and Medicines & Healthcare Products Regulatory Agency London UK; ^38^ Department of Molecular Toxicology German Institute of Human Nutrition, Potsdam‐Rehbrücke Nuthetal Germany; ^39^ Institute of Nutritional Sciences, Dept of Food Biofunctionality University of Hohenheim Stuttgart Germany; ^40^ Department of Molecular Genetics, Erasmus MC Cancer Institute, Erasmus University Medical Center Rotterdam the Netherlands; ^41^ Princess Maxima Center for Pediatric Oncology, Oncode Institute Utrecht the Netherlands; ^42^ University of Cologne, Faculty of Medicine, Cluster of Excellence for Aging Research Institute for Genome Stability in Ageing and Disease Cologne Germany; ^43^ Department of Experimental, Diagnostic and Specialty Medicine (DIMES) University of Bologna Bologna Italy; ^44^ Department of Computer Science University of Konstanz Konstanz Germany; ^45^ KNIME AG Zurich Switzerland; ^46^ German Center for Diabetes Research (DZD) München‐Neuherberg Germany; ^47^ German Center for Cardiovascular Research (DZHK), Partner Site Berlin Berlin Germany; ^48^ University of Potsdam, Institute of Nutritional Science Nuthetal Germany; ^49^ University of Vienna, Department of Physiological Chemistry, Faculty of Chemistry Vienna Austria; ^50^ Laboratory of Systems Medicine of Healthy Aging, Institute of Biology and Biomedicine and Institute of Information Technology, Mathematics and Mechanics, Department of Applied Mathematics, N. I. Lobachevsky State University Nizhny Novgorod Russia

**Keywords:** biochemical markers, biological age score, human

## Abstract

Biomarkers of ageing are defined as age‐related changes in body function or composition that could serve as a measure of ‘biological’ age and predict the onset of age‐related diseases and/or residual life expectancy. We conducted the MARK‐AGE Study, a European population study (3300 subjects aged 35–74) to identify a powerful set of biomarkers of ageing. A total of 362 clinical‐chemistry, genetic, cellular or molecular biomarkers were analysed for each subject. Using statistical models as well as machine learning we derived mathematical formulas for females and for males that yield a ‘bioage score’ of an individual, based on sets of 10 biomarkers for females and 10 for males. Collectively, these biomarkers model chronological age of our study population and, thus yield the ‘biological’ age of a certain person. ‘Age difference’ (defined as biological minus chronological age) should then identify biologically older or younger individuals. Using our set of biomarkers, subjects with Down Syndrome and smoking females are biologically older, whereas postmenopausal females taking hormone replacement therapy are biologically younger. Strikingly, our data reveal that age difference of MARK‐AGE subjects, but not chronological age, is linearly correlated with levels of HDL, 25‐hydroxy‐Vitamin D, and CD3+ CD4+/CD45+ ratio in such a way that biologically younger subjects display values that are favourable to good health, whereas other markers such as glucose and HbA1c are correlated with chronological age, but not age difference. This dichotomy of correlations may point to different roles of such markers, that is, drivers of the ageing process versus bystanders of ageing.

## Graphical abstract

Using sets of biomarkers of human ageing, a ‘bioage’ score was established allowing calculation of ‘age difference’ (bioage minus chronological age). Age difference, but not chronological age, was correlated with levels of HDL, Vitamin D, and CD3+ CD4+/CD45+ ratio, with biologically younger subjects displaying favourable values, pointing to a role of these markers as drivers of ageing.

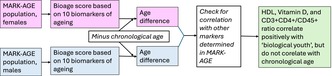

## 1 Introduction

Ageing is commonly defined as time‐dependent accumulation of diverse deleterious changes occurring in cells and tissues that are associated with and probably causative for the increased risk of morbidity and mortality (Harman 2003). Quantitative biomarkers of ageing could be important tools for determining the rate of the ageing process even in healthy individuals. Furthermore, biomarkers of ageing might serve to identify individuals at high risk of developing age‐associated disease or disability. The MARK‐AGE Consortium has conducted a systematic population study of about 3300 subjects (Baur, Kotter, et al. 2015; Baur, Moreno‐Villanueva, et al. 2015; Burkle et al. 2015; Capri et al. 2015; Giampieri et al. 2015; Jansen et al. 2015; Moreno‐Villanueva, Capri, et al. 2015; Moreno‐Villanueva, Kotter, et al. 2015) from 8 European countries, aiming to identify a battery of biomarkers of ageing that could indeed serve as a measure of biological age.

The major theories of ageing are focused on a particular cause of ageing, respectively, for example, the free radical theory, the immunologic theory, known as inflammaging (Franceschi et al. 2000; Franceschi, Capri, et al. 2007), the inflammation theory or the mitochondrial theory (Tosato et al. 2007). However, ageing is an extremely complex, multifactorial process that affects all tissues and organs of the body, and there is clear evidence that the rate of ageing differs significantly between members of the same animal species, including humans. In other words, ‘biological age’ may well differ from chronological age.

In a number of biological model systems, mutants have been isolated with either longer or shorter lifespan than wild‐type, suggesting that longevity has a strong genetic component. It is estimated, however, that in human beings genetic variation can account for at most 25% of the variability of the lifespan (Herskind et al. 1996). Therefore, other factors have to play a role. While some environmental factors, such as exposure to chemicals or radiation or chronic infection, can accelerate ageing, others, such as resveratrol or vitamin D, have been reported to slow the ageing process. In addition, dietary restriction (Minor et al. 2010) increases life expectancy in model systems (Baur et al. 2006; Jia et al. 2004; Kaeberlein et al. 1999, 2005; Kapahi et al. 2004; Masoro 2006; Rogina and Helfand 2004; Selman and Withers 2011; Tissenbaum and Guarente 2001; Weindruch 1996), and this is very likely to hold true for humans as well. Likewise, regular physical activity (Reimers et al. 2012) increases life expectancy.

Although ageing per se is a physiological phenomenon, it is also a very important risk factor for a wide variety of chronic and debilitating diseases (Niccoli and Partridge 2012). Quantitative biomarkers of ageing could be important tools for determining the rate of the ageing process even in healthy individuals.

As mentioned above, biomarkers of ageing might serve to identify individuals at high risk of developing age‐associated disease or disability. This could trigger follow‐up examinations and, if available, prophylactic intervention or early‐stage treatment of age‐related disease. Furthermore, powerful biomarkers would allow the assessment of the efficacy of outcome measures in trials of interventions, including dietary strategies or physical activity, designed to extend health span.

In order to determine biological age, previous studies have either relied on age‐associated biomarkers such as parameters measured in blood or its components (Banerjee et al. 2011; Catera et al. 2016) or physical parameters, for example, blood pressure, heart rate, or lung function (Belsky et al. 2015; Christou and Seals 2008; Fleg et al. 2005; Pinto 2007; Xu et al. 1995).

However, conventional clinical chemistry parameters on their own do not reflect the specific changes occurring during ageing at the molecular level. Furthermore, many candidate biomarkers of ageing have been proposed, but in all cases their variability in cross‐sectional studies is considerable, and therefore no single measurement has so far proven to yield a useful biomarker of ageing on its own, probably due to the multi‐causal and multi‐systemic nature of ageing (Burkle et al. 2015). In addition, any assessment of biological age that requires full cooperation and motivation of the person to be tested is prone to errors and labour‐intensive, which restricts their practical use at a wider scale.

A more recent addition to the list of biomarkers of ageing is changes in cytosine methylation (5‐methyl cytosine, 5mC) at CpG sites in DNA. 5mC is a key component of epigenetic regulation. Changes in cytosine methylation are associated with chronological age (Hannum et al. 2013; Lin et al. 2016; Xiao et al. 2019), and therefore DNA methylation has been used in recent years to estimate ‘biological age’ and to predict life and health span using various approaches (Chen et al. 2016; Levine et al. 2018; Lin et al. 2016; Lu et al. 2019; Zhang et al. 2017). ‘DNA methylation clocks’ are estimators built from epigenetic DNA methylation marks that strongly correlate with chronological age. A number of DNA methylation‐based ageing clocks have been proposed, and their individual strengths and weaknesses have been discussed (Bell et al. 2019; Field et al. 2018). For instance, DNA methylation clocks that also incorporate biochemical markers such as PhenoAge and GrimAge may be better predictors of mortality (Bell et al. 2019). However, because the methylation clocks have been developed using different biobanks and databases of specific cohorts, there is a lack of standardisation, and the cohorts may not be representative of a large population. Furthermore, in most cases, epigenetic clocks models are inconsistent with each other (Yusipov et al. 2024).

The MARK‐AGE Consortium has conducted a systematic population study of about 3300 subjects (Burkle et al. 2015; Capri et al. 2015; Moreno‐Villanueva, Capri et al. 2015) from 8 European countries, aiming to identify a battery of biomarkers of ageing that could indeed serve as a measure of biological age. Specifically, the strategy underpinning the MARK‐AGE study attempted to model the age status in a test population by a set of molecular or biochemical markers. Applying this modelling to some individual outside the reference population would yield the expected age of this person. A value of the expected age that is higher than the chronological age of the person would indicate an advancement of biological age and a value lower than chronological age a delay in biological age. A wide range of candidate biomarkers were systematically tested (i) in the RASIG subgroup (Randomly recruited Age‐Stratified Individuals from the General population) as well as in additional subgroups, comprising (ii) subjects born from a long living parent (GO), (iii) spouses of GO (SGO) and (iv) subjects with Down Syndrome (DS) (Baur, Kotter, et al. 2015; Baur, Moreno‐Villanueva, et al. 2015; Jansen et al. 2015; Moreno‐Villanueva, Kotter, et al. 2015). Data on several of these individual biomarkers have been published (Borelli et al. 2015; Ciccarone et al. 2016, 2018; Dunston et al. 2012; Ghezzo et al. 2014; Giacconi et al. 2018; Gradinaru et al. 2015; Horvath et al. 2015; Malavolta et al. 2015; Moreno‐Villanueva and Burkle 2019; Pinchuk et al. 2019; Rietman et al. 2019; Sikora 2015; Stuetz et al. 2016; Valentini et al. 2016; Vanhooren et al. 2015; Weber et al. 2017; Weinberger et al. 2018; Zampieri et al. 2010, 2015). Our statistical analyses yielded a set of 10 molecular or biochemical biomarkers for females and a set of 10 for males, as well as respective algorithms, which allowed modelling the chronological age in the study population.

## 2 Methods

### 2.1 Study Design and Participants

Details of the design of the MARK‐AGE study, including strategic and logistic aspects of subject recruitment, Standard Operating Procedures, the phenotypic database, data management, quality control of biological samples, as well as relevant aspects of some classes of biomarkers have been published (Burkle et al. 2015; Capri et al. 2015; Moreno‐Villanueva, Capri, et al. 2015; Moreno‐Villanueva, Kotter, et al. 2015). This study was conducted in accordance with the Declaration of Helsinki (1964) and with informed written consent of each participant. Ethical clearance had been given by the ethics committee of each of the recruiting centres. This study has been registered retrospectively at the German Clinical Trials Register (DRKS00007713).

In the context of the MARK‐AGE study, a total of 362 molecular, clinical chemistry, genetic and cellular biomarkers were tested for each individual, including ‘classical’ ones, for which a correlation with age had already been known, ‘new’ ones that were based on recent findings, plus parameters that were based on recent research on mechanistic aspects of ageing, conducted by members of the MARK‐AGE consortium (Burkle et al. 2015; Capri et al. 2015). All analyses were performed on coded samples as described before (Moreno‐Villanueva, Capri, et al. 2015).

### 2.2 Statistical Analysis

Machine learning, data stratification and k‐means cluster analysis were performed using KNIME software (Dietz and Berthold 2016). Outliers were identified using the interquartile range with *k* = 1.3. D'Agostino and Pearson test and Shapiro test were used for testing normality. Statistical analyses and preparation of graphs were done using GraphPad Prism 10.

Statistical analysis was performed in order to extract a robust set of biomarkers of human aging from the large amounts of data obtained. This was done by using a visual software interface (graphical workbench) for the entire analysis process, including data retrieval from our databases, data transformation, initial investigation, powerful predictive analytics, visualisation and reporting (Baur, Kotter, et al. 2015; Baur, Moreno‐Villanueva, et al. 2015). Learning was performed on the RASIG subgroup excluding women undergoing hormone replacement therapy (HRT). The analyses were conducted separately for males and females. Subjects who did not have the completed set of biomarkers assessed and subjects with an outlier biomarker ([0.01, 0.99]) were excluded from analyses. Non‐parametric effect size was calculated by Hodges‐Lehmann estimator and is reported with the confidence intervals (CI) in the captions of the corresponding figures. For linear regressions size effect estimation is reported in each plot (equation, *R* ^2^ and *p* value).

## 3 Results

### 3.1 Bioage Score

As a first step, the correlation between each biomarker measured and age was determined, resulting in (i) a parametric coefficient of correlation (rp), (ii) a non‐parametric coefficient of correlation (rnp), (iii) the slope of the linear regression curve (a) and (iv) the intercept of the linear regression curve (c). This was done separately for female and male subjects, since sex differences in the biomarkers were expected. The non‐parametric coefficient of correlation (rnp) for the *z*‐scaled biomarkers used for the bioage formula are shown in Table S1, and the respective graphs are shown in Figures S1–S20.

In order to aggregate several age‐correlating biomarkers into some overall predictor of the age state, a so‐called biological age, we started from a population Ω with variables A and M describing the chronological age and a vector of selected biomarkers for each individual. A natural definition of a biological age $\tilde{B}$ (relative to the given population) would start from the markers M of a given individual, collect all members of the population with similar marker values and take their average biological age. Mathematically, this amounts to the definition $\tilde{B}=E\left(A|M\right)$, that is, $\tilde{B}$ is the conditional expectation of chronological age given the marker values. Since the conditional expectation can be computed as best‐approximation of A within a suitable space of M‐dependent functions, this leads to a least‐squares approximation problem. As a numerical approximation, we use the space of *affine linear* functions (i.e., linear functions plus a constant shift that preserve straight lines but does not have to pass through the origin) in M and end up with a linear regression problem.

In practical terms, 30 parameters that had been preselected for the best (positive or negative) correlation with chronological age in women and men, respectively, of the RASIG subpopulation were fed into the Leaps package in the software package R for regression subset analysis. An algorithm incorporated in the Leaps package selected the 10 most suitable parameters. Using the Stats package, a *Z*‐score scaling was applied to these 10 parameters and a multiple linear regression model was built, resulting in a weighting factor for each scaled biomarker (weighting factors b_1_–b_10_), a correlation coefficient *r* ^2^ for the multiple linear regression, and an intercept c for the multiple linear regression. Based on these data, the (raw) biological age of any individual subject can be calculated according to the formula:

$$\tilde{B}=\sum_{i=1}^{10}b_{i}z_{i}+c$$

where $\tilde{B}$ is the raw biological age, *z*_1_ through *z*_10_ are the scaled values of *x*_1_ through *x*_10_ which are the values of the respective biomarkers measured. In this formula the weighting factors *b*_1_–*b*_10_ (in short: *b*_i_) determine the strength of the relative contribution of each marker to the raw biological age. The *Z*‐score scaling (also called *Z*‐score normalisation) is used to transform data into a standard normal distribution, ensuring that all biomarkers are on the same scale, and is defined according to the rule

$$z_{i}=\frac{x_{i}-{\bar{x}}_{i}}{\sigma_{i}}$$

wherein ${\bar{x}}_{i}$ is the arithmetic mean and $\sigma_{i}$ the standard deviation of biomarker *i* in the RASIG population.

In order to facilitate usage of the biological age values, a strictly monotone transformation was applied to the raw biological age which ensures that the average biological age approximately equals the chronological age in randomly selected even‐aged groups of individuals while not disturbing the biologically‐younger‐than relation resulting from $\tilde{B}$. The transformation is given by the formula

$$B=\frac{\tilde{B}-\text{mean}\;\left(a\right)}{r^{2}}+\text{mean}\;\left(a\right)$$

wherein B is the (corrected) biological age score (also termed bioage score); ‘mean (*a*)’ is the mean age of the whole RASIG population; and ‘*r*’ is the correlation coefficient for the correlation between chronological age and $\tilde{B}$ in the population. Values for ${\bar{x}}_{i}$, $\sigma_{i}$, *b*_i_, c and *r* ^2^ for females and for males are provided in Tables S1 and S2.

Extensive bioinformatic analysis was performed in order to extract a robust set of biomarkers of chronological ageing from the large amount of data obtained (Baur, Kotter, et al. 2015; Baur, Moreno‐Villanueva, et al. 2015). We identified a set of 10 biomarkers for females ‘F1’ through ‘F10’ and also 10 for males ‘M1’ through ‘M10’ (Table 1; Supporting Information; Figures S1–S13) that allowed derivation of a bioage score with a correlation coefficient with chronological age of Pearson *r* = 0.8976 for females and *r* = 0.8741 for males (Figure 1A,B).

**TABLE 1:** Overview of the biomarkers used in the bioage algorithms for females and males, respectively, and their correlation (rnp) with chronological age.

#	Biomarker designation	Marker #(f)	Figure (suppl. data)	Marker #(m)	Figure (suppl. data)	Count female	rnp female	Count male	rnp male
1	ELOVL2 CpG 11.12.13.14	F1	S 1	M1	S 11	888	0.796	842	0.758
2	ELOVL2 CpG 15.16.17	F2	S 2	M2	S 12	888	0.775	842	0.732
3	FHL2 CpG 11.12	F3	S 3	M3	S 13	888	0.463	842	0.450
4	FHL2 CpG 13.14.15	F4	S 4	M4	S 14	888	0.560	842	0.552
5	FHL2 CpG 16.17	F5	S 5	M5	S 15	888	0.598	842	0.534
6	Dehydroepiandro‐sterone sulphate	F6	S 6	M6	S 16	888	−0.480	842	−0.561
7	S p6 n glycan	F7	S 7	M7	S 17	888	−0.654	842	−0.301
8	Log ferritin	F8	S 8	—	—	888	0.457	—	—
9	S log p1/p6	F9	S 9	—	—	888	0.640	—	—
10	Plasma α‐tocopherol	F10	S 10	—	—	888	0.336	—	—
11	α 2‐macroglobulin	—	—	M8	S 18	—	—	842	0.287
12	Lycopene	—	—	M9	S 19	—	—	842	−0.367
13	Log prostate specific antigen	—	—	M10	S 20	—	—	842	0.292

*Note:* Biomarker #(f): identifier of biomarker for females. Biomarker #(m): identifier of biomarker for males. Fig.: biomarker correlation data shown in Supporting Information Figure. Count female/male: number of RASIG females/males analysed. rnp female/male: non‐parametric correlation coefficient for females/males.

**FIGURE 1:**
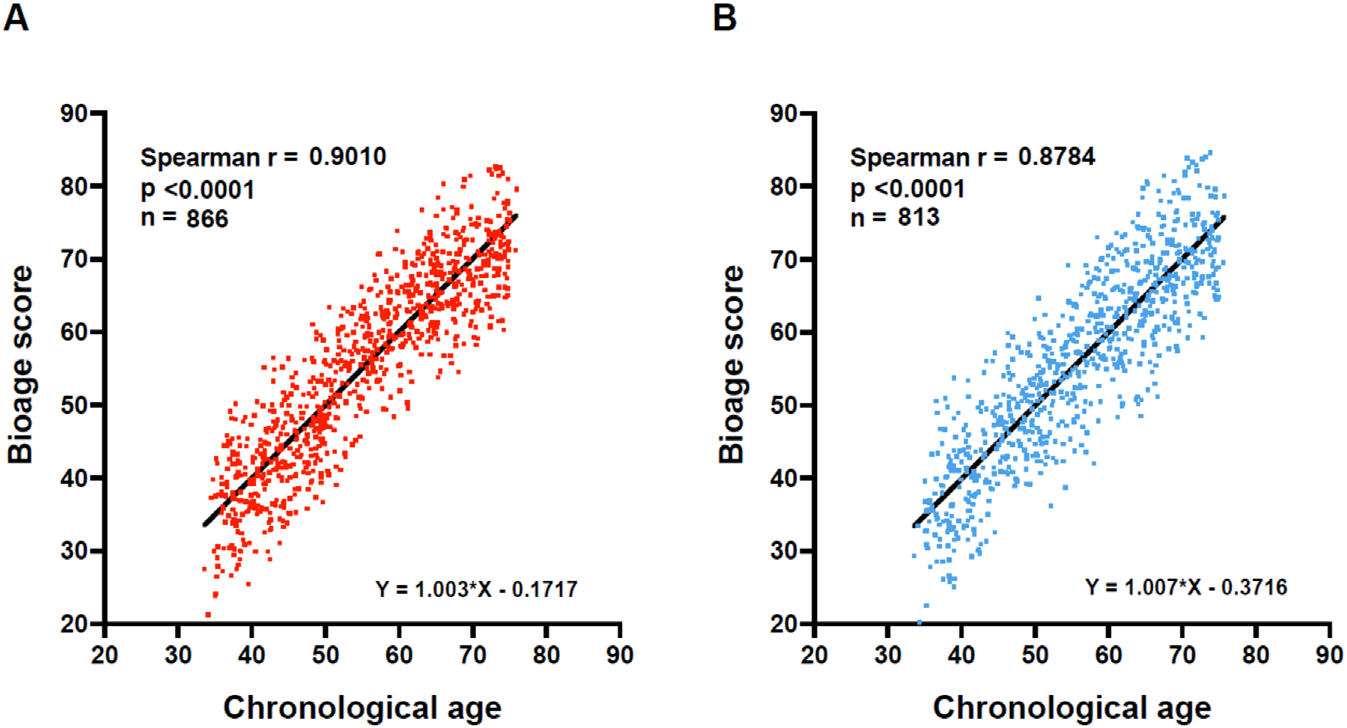
Scatter plots representing the correlation between chronological age (*x*‐axis) and the calculated bioage score (*y*‐axis) in females (A) and males (B) of the RASIG subgroup.

Collectively, the bioage scores of the members of the MARK‐AGE population model chronological age of these individuals via molecular and clinical chemistry analyses. The bioage score of an individual outside the RASIG group will yield the ‘predicted chronological age’ of this person, and we define this value as the person's ‘bioage’ (biological age). The numerical difference between bioage and chronological age then yields a measure for the ‘age difference’ of this individual: either ‘age advancement’ (if the numerical value is positive) or ‘age delay’ (if negative).

To check whether age difference as determined by using the MARK‐AGE bioage score indeed reflects known or expected differences in biological age, we analysed the effects of Down syndrome (DS), cigarette smoking, and hormone replacement therapy (HRT) in postmenopausal women.

### 3.2 Age Difference of Down Syndrome Subjects

DS individuals may exhibit some features of accelerated ageing, and therefore DS is considered a progeroid syndrome. DS individuals and their families were contacted by the MARK‐AGE team at the University of Bologna, Italy. A total of 52 DS individuals were successfully recruited, with a specific strategy committed to family involvement and the involvement of health care professionals, such as psychologists and psychiatrists (Capri et al. 2015). Fifteen DS subjects did not complete the required measurements for the calculation of the biological age, and therefore only data from 37 DS subjects were included in the analysis. These 37 subjects also comprised a number of young individuals (7 females and 7 males) outside the MARK‐AGE age range. In order to compare DS with age‐matched RASIG subjects, the younger subjects were first excluded from the analyses. DS females (Figure S23A) and males (Figure S23B) displayed significantly higher age difference (i.e., bioage score minus chronological age) than the age‐matched RASIG population. The age difference values from the complete set of DS subjects analysed are listed in Tables S3 and S4. Mean age difference and the result of statistical testing of the biological vs. chronological age of a person are also shown. The data reveal a mean age advancement of 5.06 (±7.12) years for females and 3.9 (±6) years for males. This is in perfect agreement with the accelerated ageing process in DS (cf. Borelli et al. 2015; Ciccarone et al. 2018; Ghezzo et al. 2014; Horvath et al. 2015).

### 3.3 Age Difference of Smokers

Several recent studies suggest that cigarette smoking can accelerate the ageing process (Astuti et al. 2017; Banks et al. 2015; Gao et al. 2016; Lei et al. 2017; Mamoshina et al. 2019). Our results from current smokers indeed show a significant positive correlation between cumulative number of cigarettes smoked (reported as number of packs smoked until the time of enrolment) and biological age in women (Figure S24A). This effect is in line with the above‐mentioned publications, and the apparent ‘dose‐dependency’ further supports the presumed causality of smoking in the acceleration of ageing. No significant difference, however, could be observed in males (Figure S24B).

### 3.4 Effect of Hormone Replacement Therapy on Biological Age

HRT in postmenopausal women is known to counteract some ageing‐associated changes, despite the presence of adverse effects of HRT (Rinaldi 2004; Samaras et al. 2014; Sites 2008). In the MARK‐AGE population, no significant difference between hormonal use and non‐use was found in women younger than 50 years (Figure S25A). But strikingly, women older than 50 years (i.e., postmenopausal) taking hormones were biologically younger by 2.1 years (unpaired *t*‐test, *p* = 0.0001) than RASIG females who were non‐users (Figure S25B). Most likely, the women under 50 took hormones for contraception, whereas those over 50 took them as HRT. Once again, our data are in full agreement with the above‐mentioned publications on HRT as an intervention counteracting some ageing‐associated changes.

### 3.5 Age‐Difference in Individuals From Long‐Lived Families and Their Spouses

Individuals born from a long‐living parent belonging to a family with long‐living sibling(s) had already been recruited in the framework of the GeHA project (Franceschi, Bezrukov, et al. 2007) (GeHA Offspring; GO) and were assumed to display a favourable biochemical profile and therefore are predicted to age at a slower rate than the average population (RASIG). As in several previous studies on this topic, spouses of GO (SGO) have been recruited in parallel. as an additional lifestyle control group. Due to genetic advantage, it was expected that GO would display a lower biological age than SGO. However, there were no significant differences in age advancement between the two groups in the MARK‐AGE cohort (Figure S21).

### 3.6 Biomarkers Associated With Age Advancement

**FIGURE 2:**
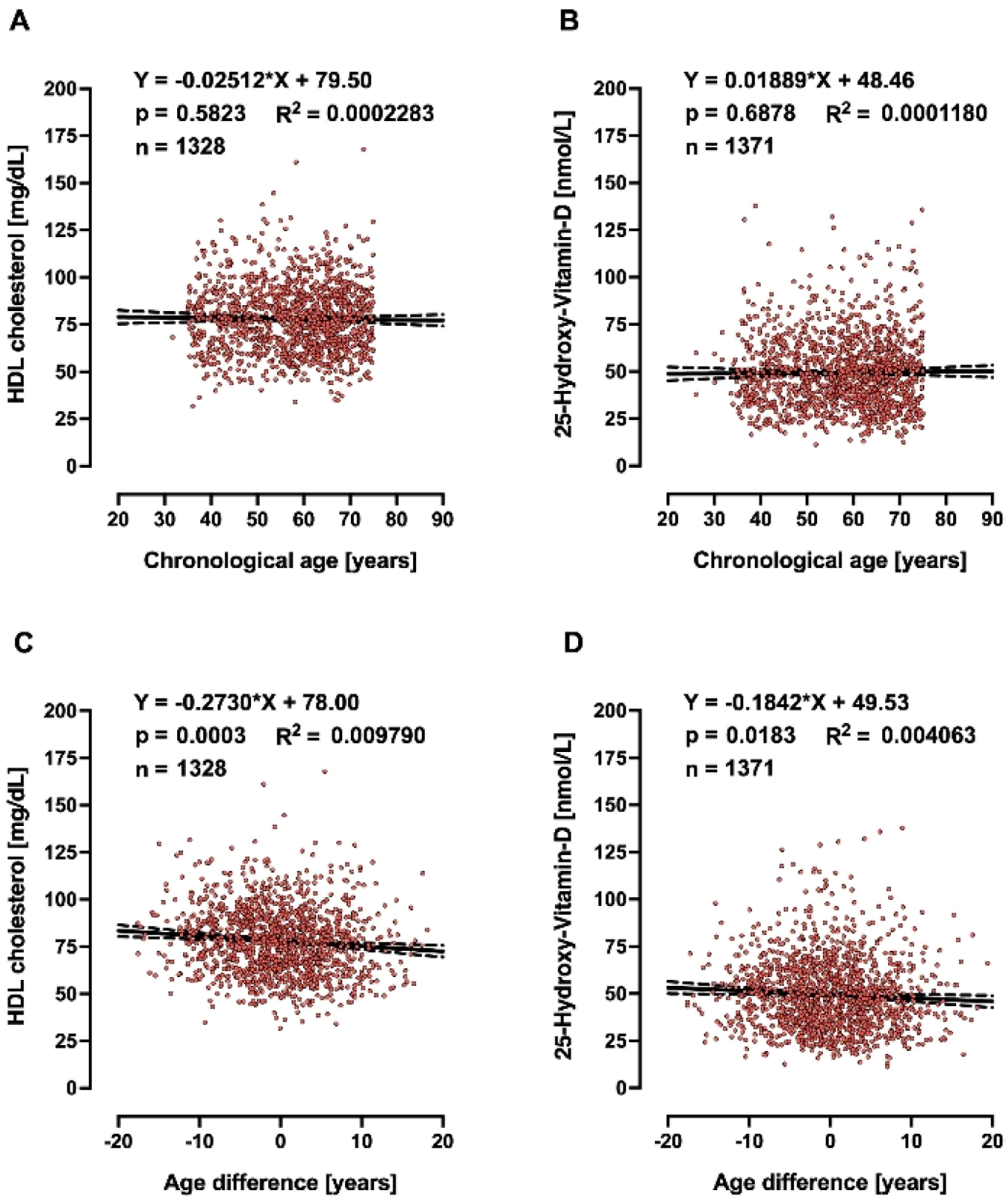
Linear regressions of chronological (A, B) and age difference (C, D) on HDL and 25‐hydroxy‐VitD in females.

**FIGURE 3:**
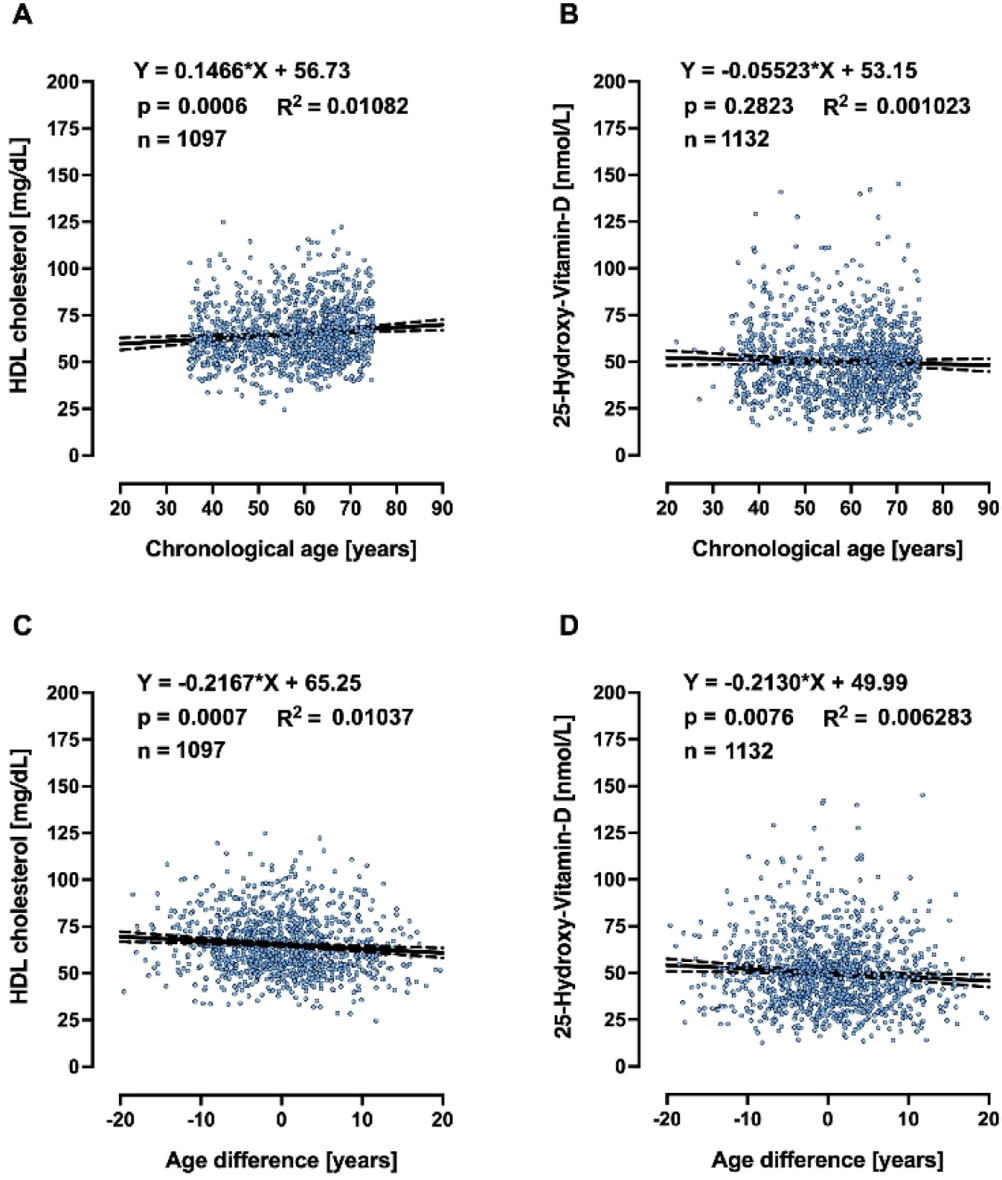
Linear regressions of chronological age (A, B) and age difference (C, D) on HDL and 25‐hydroxy‐VitD in males.

**FIGURE 4:**
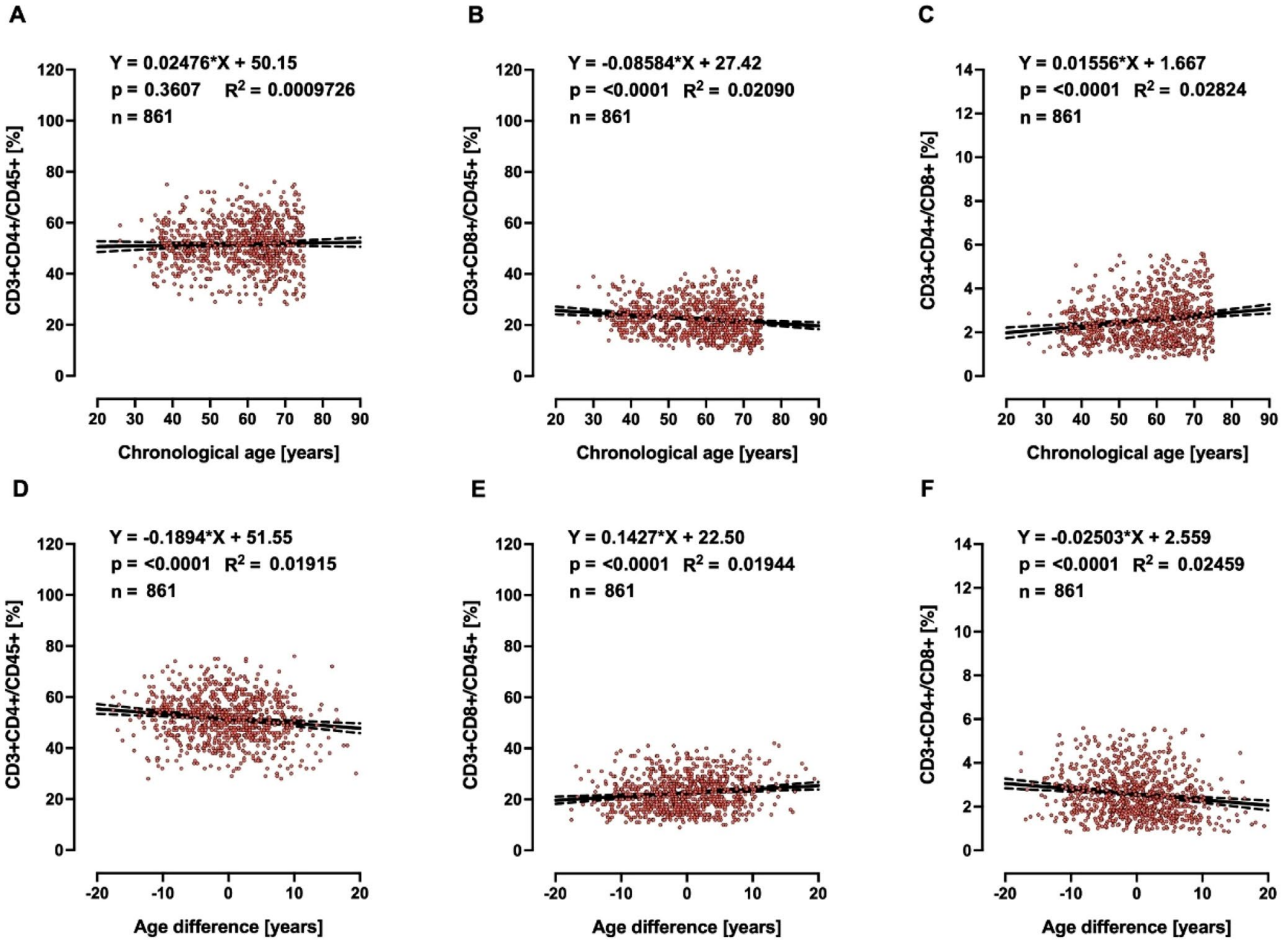
Linear regressions of chronological age (A, B, C) and age difference (D, E, F) of CD4+ T and CD8+ T cells in females.

**FIGURE 5:**
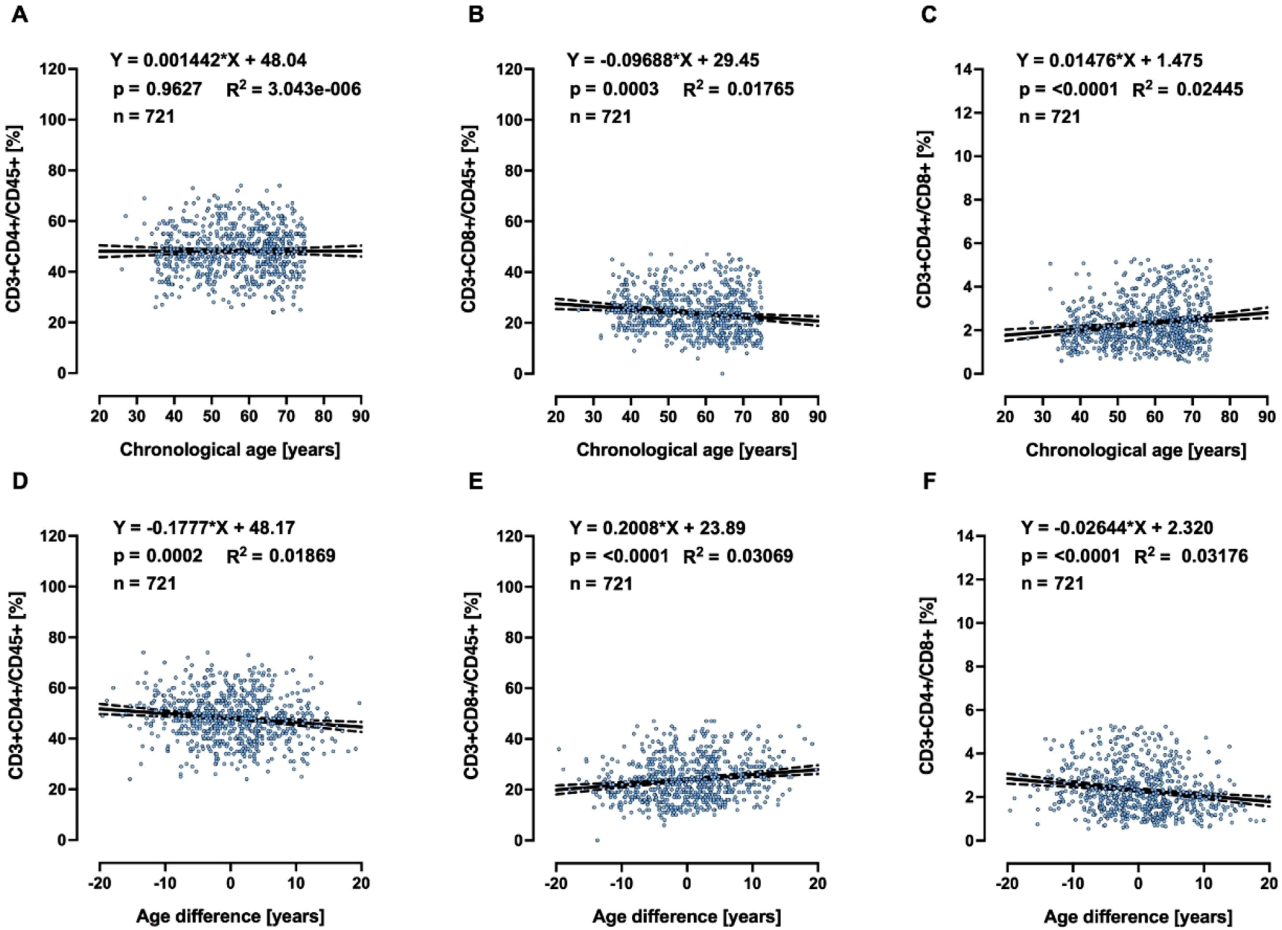
Linear regressions of chronological age (A, B, C) and age difference (D, E, F) of CD4+ T and CD8+ T cells in males.

A biomarker of ageing is a biological parameter that—either alone or in some multivariate composite—will better predict functional capability than chronological age. Therefore, biological age should outperform chronological age in predicting the pace of ageing, since individuals with the same chronological age can vary in health. Based on this, we searched for clinically relevant markers, in the whole collection of measurements performed in MARK‐AGE, that would be associated with age advancement, but not with chronological age, and identified 25‐hydroxy‐VitD, HDL (Figures 2 and 3 for females and males, respectively) and T cell subpopulations (Figures 4 and 5 for females and males, respectively). It is striking to see that age difference is linearly correlated with levels of HDL, 25‐hydroxy‐Vitamin D and CD3+ CD4+/CD45+ ratio, in such a way that biologically younger subjects display values that are considered favourable to good health.

### 3.7 Biological Age and Glucose Homeostasis

**FIGURE 6:**
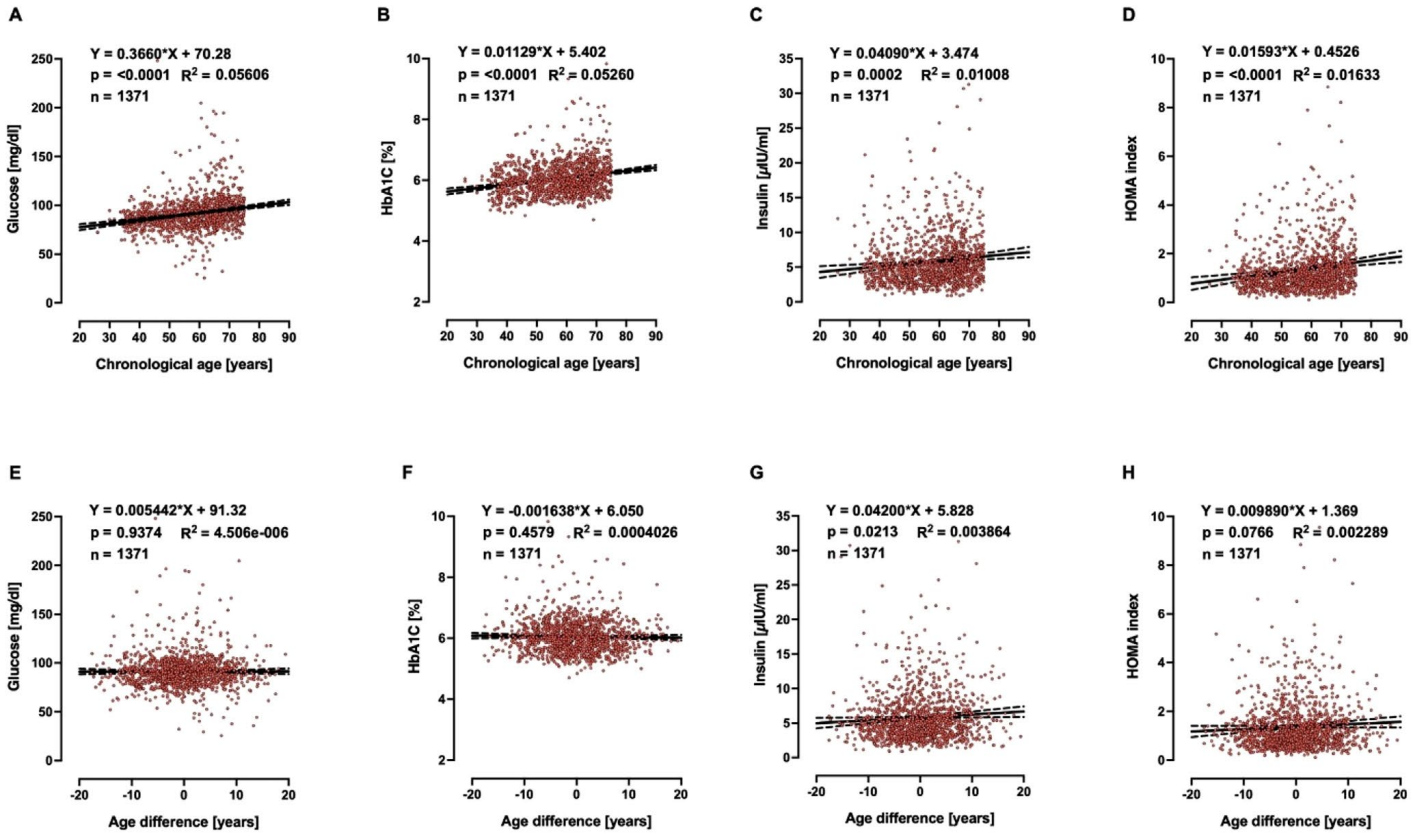
Linear regressions of chronological age and age difference on fasting glucose, HbA1c, fasting insulin and HOMA index in females. (A, E): Glucose; (B, F) HbA1C; (C, G): Insulin; (D, H): HOMA index.

In our search for correlations between age difference and routine clinical chemistry markers associated with disease risk, which are not incorporated in the female or male MARK‐AGE bioage scores, we were also interested in glucose homeostasis, in view of several studies showing that one common feature of ageing is the progressive decline in the accurate regulation of glucose homeostasis (Kurauti et al. 2021; Tuduri et al. 2022). Therefore, we asked whether age advancement would be associated with high levels of glucose or glycosylated haemoglobin A_1c_ or insulin. Glucose concentrations above 250 mg/dL are considered severe hyperglycaemia, and therefore the subjects (*n* = 5) with glucose higher than 250 mg/dL were excluded from analyses. Chronological age is associated with high levels of serum glucose and glycosylated haemoglobin A_1c_ in females (Figure 6A,B) and males (Figure S26A,B). Interestingly, age difference, but not chronological age, is associated with insulin plasma concentration in males (Figure S26G,H), while in females, both chronological age (Figure 6C,D) and age difference (Figure 6G,H) are associated with insulin plasma concentrations and insulin resistance as assessed by HOMA index. For clarity, the slopes and *p*‐values from Figures 2, 3, 4, 5, 6 and Figure S26 are summarised in Table 2.

**TABLE 2:** Slopes and *p*‐values from graphs shown in Figures 2, 3, 4, 5, 6 and S26.

Marker as a function of		Females		Males	
		Slope	*p*	Slope	*p*
HDL	CA	−0.0251	0.5823	0.1466	**0.0006**
	AD	**−0.2730**	**0.0003**	**−0.2167**	**0.0007**
Vit‐D	CA	0.0188	0.6878	−0.0552	0.2823
	AD	**−0.1842**	**0.0183**	**−0.2130**	**0.0076**
CD3+ CD4+/CD45+	CA	0.0247	0.3607	0.0014	0.9627
	AD	**−0.1894**	**< 0.0001**	**−0.1777**	**0.0002**
Glucose	CA	**0.3660**	**< 0.0001**	**0.3814**	**< 0.0001**
	AD	0.0054	0.9374	0.0786	0.2920
HbA1c	CA	**0.0112**	**< 0.0001**	**0.0088**	**< 0.0001**
	AD	0.0016	0.4579	0.0015	0.5191
Insulin	CA	**0.0409**	**0.0002**	−0.0099	0.4804
	AD	**0.0420**	**0.0213**	**0.0894**	**< 0.0001**
HOMA	CA	**0.0159**	**< 0.0001**	0.0043	0.2842
	AD	0.0098	0.0766	**0.0894**	**< 0.0001**

*Note:* The higher (absolute) values of slope in the chronological age (CA) versus age difference (AD) comparisons as well as all significant *p*‐values are shown in boldface.

## 4 Discussion

In the MARK‐AGE study, we have focussed on molecular and biochemical parameters in peripheral blood as potential biomarkers of human ageing. By using statistics and machine learning algorithms we have identified sets of 10 molecular biomarkers for women and 10 for men that can be used for estimating the biological age of an individual. It is striking that in the MARK‐AGE population, age difference (calculated as MARK‐AGE bioage score minus chronological age) is linearly correlated with levels of HDL, 25‐hydroxy‐Vitamin D, and CD3+ CD4+/CD45+ ratio in females and males, in such a way that biologically younger subjects display values that are considered favourable to good health. Importantly, there is a remarkable selectivity in this observation, as glucose levels, for instance, are *not* correlated with age difference, but with chronological age.

When establishing the MARK‐AGE ‘bioage score’, it was remarkable to see the relatively high variability of biological age in subjects with the same chronological age, which is perfectly in line with the notion that everyone has their own biological ageing process. This highlights the central role of personalised/precision medicine. Thus, biological age at individual level may pave the way for such approaches in a relatively simple manner (Franceschi et al. 2017). Viewed together, the availability of powerful biomarkers of ageing could represent an enormous step forward in the context of preventive medicine.

In order to perform a set of plausibility checks for the validity of our biomarkers of ageing, we verified if expected differences in biological age in certain groups of individuals are indeed detectable by using our sets of biomarkers. The groups we selected were (i) DS individuals, (ii) smokers and (iii) postmenopausal women taking HRT. We found in individuals with DS that their average biological age is indeed higher than the average biological age of the general population. These data are in line with previous studies. Horvath's clock (DNA methylation of 353 CpG sites) revealed that in DS individuals, blood and brain tissue age is significantly increased (on average by 6.6 years) (Horvath et al. 2015). Furthermore, a study on 76 DS subjects, 37 siblings and 42 mothers of DS subjects identified specific glycomic features in line with accelerated ageing (Borelli et al. 2015).

In our analyses, biological age also differed from chronological age in two other subgroups of individuals, that is, smokers and postmenopausal women taking hormones. Regarding smokers, we considered current smokers only, with their quantitative smoking history expressed as cumulative numbers of packs of cigarettes smoked until the time of enrolment. Our results show a significant association between the cumulative number of cigarettes smoked and age difference in females, but not in males. Interestingly, data from the literature suggest that, compared to men, women may be more susceptible to smoking‐related morbidity and mortality (Allen et al. 2014). More recent studies report that women are more vulnerable to smoking regarding earlier death and risk of stroke, but less vulnerable regarding lung disorders in older people (Haghani et al. 2020). Furthermore, using routine clinical chemistry analysis, female smokers were found to show higher biological age than male smokers (Mamoshina et al. 2019). Thus, our data support previous work showing the individual's ageing rate might be accelerated by smoking and that this effect might be sex‐dependent.

Regarding hormone intake, women were asked the question whether they have taken hormones during the last year. Unfortunately, we cannot distinguish between contraceptive uses versus hormone replacement therapy (HRT), since this specific question was not included in the MARK‐AGE questionnaires. However, women older than 50 usually have entered the phase of menopause and therefore are more likely to have received HRT, while younger women are more likely to have used contraceptive pills. Our results demonstrate that women older than 50 who answered this question with ‘yes’ are biologically younger, suggesting a slower ageing rate for this particular group on average. Indeed, the anti‐ageing effects of HRT have been discussed. However, long‐term oestrogen and progesterone intake might also have adverse effects including cancer development (Rinaldi 2004; Samaras et al. 2014; Sites 2008). Nevertheless, women report that they experience an anti‐ageing efficacy of HRT despite medical risks (Hunter et al. 2020). On the other hand, Hodis and collaborators summarised evidence for beneficial effects of menopausal HRT when initiated in close proximity to menopause but also reassurance of long‐term safety (Hodis and Mack 2022). Unfortunately, the MARK‐AGE study cannot provide further information, since participants were not asked the question for how long they had been taking hormones.

In line with data from the literature (Ghasemi et al. 2011; Ko et al. 2006), we found a positive correlation between chronological age and fasting glucose and with glycosylated haemoglobin in males and females. Regarding insulin concentration, however, a study reported no change of fasting insulin with age in subjects between 65 and 85 years old (Lindberg et al. 1997), while a more recent study on individuals between 24 and 78 years found a significant decline of insulin concentration with advancing age in men, but not in women (Bryhni et al. 2010). Furthermore, it should be noted that Yang et al. (2023) used routine blood chemistry markers, such as blood urea nitrogen, serum C‐reactive protein, glycosylated haemoglobin Hb1Ac, serum creatinine, serum alkaline phosphatase, serum albumin and serum total cholesterol, for the assessment of biological age and found that biological age increases with increasing HOMA index. However, the clinical chemistry parameters glycosylated haemoglobin, total cholesterol and CRP are closely related to glucose homeostasis. Therefore, their use for calculating biological age creates a risk of tautology, as the biological age determined was found to correlate with the HOMA index. Nevertheless, since the Clinical Chemistry work package of the MARK‐AGE project comprised almost all of the above‐mentioned clinical chemistry parameters (with the exception of alkaline phosphatase), we checked the markers proposed by Yang et al. (2023) by calculating the biological age in the MARK‐AGE subjects based on these biomarkers, and we could confirm a correlation between biological and chronological age (Figure S22).

In our search for clinically relevant markers within the whole collection of measurements performed in MARK‐AGE that would be associated with age advancement, but not with chronological age, we identified HDL cholesterol and 25‐hydroxy‐vitamin D to have this property. A predictive model of longevity has been recently published based on the data collected in individuals of the longitudinal D‐EPESE study (Kraus et al. 2022). The numbers of small HDL particles, younger age and fewer pack years of cigarette smoking were the strongest determinants of longevity (Kraus et al. 2022). Furthermore, high HDL‐cholesterol levels have been associated with lower epigenetic age acceleration in the Berlin Age Study II (Lemke et al. 2022). Regarding the association between VitD and bioage, *DNA methylation‐based age acceleration* estimated with the 7‐CpG clock, Horvath's clock, Hannum's clock, PhenoAge and GrimAge revealed that intake of vitamin D supplements is associated with lower *DNA methylation‐based age acceleration* in participants with vitamin D deficiency (Vetter et al. 2022). Interestingly, vitamin D supplementation also appeared to have beneficial effects on increasing the HDL levels in healthy adolescents (Yarparvar et al. 2020).

Ageing also induces several changes in the immune system. For instance, the total CD8+, but not CD4+, T cell numbers decrease with age while the ratio of CD4 to CD8 increases with age (Li et al. 2019). When looking at the association with chronological age we could verify these findings in the MARK‐AGE population (Figure 6 and Supplementary Figure S26A–C), but interestingly the outcome was the opposite when looking at the association with age difference. Recently, single‐cell transcriptomic studies revealed a marked increase of cytotoxic CD4 cells (CD4 CTL) as a signature of supercentenarians, who represent a ‘successful ageing’ phenotype (Hashimoto et al. 2019). Other studies proved that this particular subtype of T lymphocytes is effective in the elimination of senescent fibroblasts in the skin (Hasegawa et al. 2023). Moreover, CD4 CTL depletion in aged mice was shown to be associated with increased senescent cell load, frailty, and decreased survival (Elyahu et al. 2024) (preprint: Elyahu et al. 2024). Thus, one may hypothesise that increased CD3+ CD4+/CD45+ ratio in biologically younger individuals could decrease the number of senescent cells, that is, one of the drivers of ageing phenotype. On the other hand, an inverse (or low) CD4+/CD8+ ratio has been linked to immunodeficiency (McBride and Striker 2017). Age advancement calculated with the MARK‐AGE biological age score was detected in a HIV+ cohort using highly efficient anti‐retroviral therapy (De Francesco et al. 2019). HIV‐positive participants demonstrated greater age advancement than the HIV‐negative study participants, and CD8+ T cell count was associated with increased age advancement, independent of HIV status/group (De Francesco et al. 2019).

The correlations reported here between HDL cholesterol, 25‐hydroxy‐vitamin D and CD3+ CD4+/CD45+ ratio, respectively, with age difference are statistically significant, but it should be kept in mind that the effect sizes, as assessed by slope and *R* ^2^ values, are rather small. We therefore do not claim that each of these three parameters is by itself a major causative determinant of the rate of the ageing process and/or may have a dominant effect on the clinical aspects of ageing. Instead, we propose that each of them may make some contribution. Clearly more work, including mechanistic studies, should be done to describe their respective contributions in greater detail.

It is striking that in the MARK‐AGE population, age difference (calculated as MARK‐AGE bioage score minus chronological age) is linearly correlated with levels of HDL, 25‐hydroxy‐Vitamin D, and CD3+ CD4+/CD45+ ratio in females and males, in such a way that biologically younger subjects display values that are considered favourable to good health. For insulin and HOMA index, however, the same correlation with age difference was found for males only. By contrast, glucose and HbA1c are correlated with *chronological* age, but not age difference, in females and males. This dichotomy of correlations—either with age difference or with chronological age—is surprising and very interesting, as it could point to different roles of such markers: one group of markers could be determinants of the pace of the ageing process and/or drivers of pathology whereas the other group could be bystanders of ageing. Clearly, more work is needed to obtain the full picture of the determinants of age acceleration in humans.

It should be noted that a potential weakness of our study could be some bias resulting from the exclusion of subjects with incomplete datasets, which is, however, rather unlikely as the biological samples were shipped to the respective analytical laboratories in a coded fashion. Furthermore, our checking of the age distribution of the subjects with complete datasets failed to show any significant distortion that could lead to some selection bias (Figure S27). Likewise, in studies on human populations, potential confounding factors indeed may play a role. But since the bioage score we have constructed is based on the combination of 10 markers, respectively, a dominant effect of any potential confounding factor linked with one such marker is rather unlikely. Furthermore, it should be kept in mind that MARK‐AGE is a cross‐sectional study, and it would be highly desirable to turn it into a longitudinal study in order to assess the predictive power of the age difference that was obtained for any MARK‐AGE subject at the time of enrolment. This should be done by systematically assessing physical, cognitive and psychological status, clinical risk scores, morbidity or mortality of subjects after enrolment.

## Author Contributions

Conceptualisation: Alexander Bürkle, Claudio Franceschi, Tilman Grune, Michael Junk, Beatrix Grubeck‐Loebenstein, Eugenio Mocchegiani, Sebastiano Collino, Martijn E.T. Dollé, Valerie Vanhooren, Paola Caiafa, Bertrand Friguet, P. Eline Slagboom, Rudi Westendorp, Antti Hervonnen, Richard Aspinall, Jan H.J. Hoeijmakers, Michael R. Berthold. Methodology: María Moreno‐Villanueva, Michael Junk, Grażyna Mosieniak, Ewa Sikora, Miriam Capri, Paolo Garagnani, Nicolle Breusing, Jürgen Bernhardt, María Blasco, María Zondag, Birgit Weinberger, Simone Fiegl, Marco Malavolta, Sebastiano Collino, Efstathios S. Gonos, Daniela Gradinaru, Eugène Jansen, Michel Salmon, Peter Kristensen, Helen Griffiths, Andreas Simm, Duncan Talbot, Bertrand Friguet, Mikko Hurme, Sheila Govind, Daniela Weber, Wolfgang Stuetz, Iuliia Gavriushina, Oliver R. Sampson, Gastone Castellani. BioBank: Tilman Grune and Nicolle Breusing. Recruitment of subjects: Grażyna Mosieniak, Ewa Sikora, Miriam Capri, Jürgen Bernhardt, Christiane Schön, Florence Debacq‐Chainiaux, Beatrix Grubeck‐Loebenstein, Simone Fiegl, Efstathios S. Gonos, P. Eline Slagboom, Rudi Westendorp, Antti Hervonnen, Mikko Hurme, Claudio Franceschi. Database: Michael R. Berthold, Michael Junk, María Moreno‐Villanueva. Formal analysis and data curation: María Moreno‐Villanueva, Michael Junk, Grażyna Mosieniak, Ewa Sikora, Miriam Capri, Paolo Garagnani, Chiara Pirazzini, Nicolle Breusing, Jürgen Bernhardt, Christiane Schön, María Blasco, Gerben Zondag, Florence Debacq‐Chainiaux, Beatrix Grubeck‐Loebenstein, Birgit Weinberger, Eugenio Mocchegiani, Marco Malavolta, Robertina Giacconi, Francesco Piacenza, Sebastiano Collino, Efstathios S. Gonos, Daniela Gradinaru, Martijn E.T. Dollé, Eugène Jansen, Michel Salmon, Peter Kristensen, Helen Griffiths, Claude Libert, Valerie Vanhooren, Andreas Simm, Duncan Talbot, Paola Caiafa, Maria Giulia Bacalini, Michele Zampieri, Bertrand Friguet, Isabelle Petropoulos, P. Eline Slagboom, Rudi Westendorp, Antti Hervonnen, Richard Aspinall, Sheila Govind Daniela Weber, Wolfgang Stuetz, Iuliia Gavriushina, Oliver R. Sampson, Gastone Castellani, Michael R. Berthold, Tilman Grune, Claudio Franceschi, Alexander Bürkle. Acquisition of funding: Alexander Bürkle, Claudio Franceschi, Tilman Grune, Jan H.J. Hoeijmakers. Writing – original draft preparation: María Moreno‐Villanueva, Alexander Bürkle. Writing – review and editing: All authors. All authors have agreed on the manuscript.

## Funding

Financial support by the European Commission, via Grant HEALTH‐F4‐2008‐200880, is gratefully acknowledged.

## Conflicts of Interest

Some of the authors (Alexander Bürkle, Michael Junk, Michael R. Berthold, María Moreno‐Villanueva, Jürgen Bernhardt, María Blasco, Jan H.J. Hoeijmakers, Beatrix Grubeck‐Loebenstein, Eugenio Mocchegiani, Marco Malavolta, Sebastiano Collino, Efstathios S. Gonos, Ewa Sikora, Daniela Gradinaru, Martijn E.T. Dollé, Peter Kristensen, Helen Griffiths, Helen Libert, Tilman Grune, Claudio Franceschi, Andreas Simm, Paola Caiafa, Bertrand Friguet, Antti Hervonen, Richard Aspinall) are co‐inventors of *European Patent No. 2976433 “Method for the determination of biological age in human beings” (*Status: not‐in‐force).

## Supporting information


**Data S1:** acel70437‐sup‐0001‐Supinfo.docx.

## Data Availability

The data that support the findings of this study are available on reasonable request from the corresponding author. The data are not publicly available due to privacy or ethical restrictions.
