# Supplementary material for: Biologically Younger Individuals, as Identified by MARK‐AGE Biological Age Scores, Display a Distinct Favourable Blood Chemistry Profile Regardless of Age
Source: Aging Cell. 2026 Mar 13;25(3):e70437. doi: 10.1111/acel.70437 (PMC13093664; doi:10.1111/acel.70437)
Supplement: Supplementary file 1 — Data S1: acel70437‐sup‐0001‐Supinfo.docx. [file ACEL-25-e70437-s001.docx]

**Biologically Younger Individuals, as Identified by MARK-AGE Biological Age Scores, Display a Distinct Favourable Blood Chemistry Profile Regardless of Age**

María Moreno-Villanueva et al.

# SUPPLEMENTARY INFORMATION

# SUPPLEMENTARY TEXT

# Biomarker description

# The following provides a detailed description of the biomarkers used in the bioage algorithms for females and males, respectively (Supplementary Figures S1-S13)

I. ELOVL 2 CpG 11, 12, 13, 14

This biomarker is defined as the cumulative level of cytosine methylation at gene positions ELOVL 2 CpG 11, ELOVL 2 CpG 12, ELOVL 2 CpG 13, and ELOVL 2 CpG 14. ELOVL 2 is a gene belonging to the ELOVL (Elongation of very long chain fatty acids) gene family whose gene products control the synthesis of very-long-chain fatty acids. The ELOVL 2 gene has a number of CpG motifs in which the cytosine can be methylated or unmethylated. For this biomarker, the methylation of the CpG motifs ELOVL 2 CpG 11, ELOVL 2 CpG 12, ELOVL 2 CpG 13, and ELOVL 2 CpG 14 is determined in blood cells in a cumulative manner over all measured cells. This marker is given as a percentage between 0 and 1, i.e. when none of the above CpG motifs are methylated, the value is 0, and when e.g. all of the above CpG motifs are methylated, the value is 1.

II. ELOVL 2 CpG 15, 16, 17

In an analogous manner to the above biomarker ELVOL 2 CpG 11, 12, 13, 14, this biomarker is defined as the cumulative level of cytosine methylation at gene positions ELOVL 2 CpG 15, ELOVL 2 CpG 16, and ELOVL 2 CpG 17.

III. FHL 2 CpG 11, 12

This biomarker is defined as the cumulative level of cytosine methylation at gene positions FHL 2 CpG 11 and FHL 2 CpG 12. FHL 2 is the gene encoding the ‘Four and a half LIM domains protein 2’ (FHL2) which contains highly conserved double zinc finger motifs called the LIM domain. FHL2 has been shown to interact with a number of intracellular protein factors. Cumulative cytosine methylation is determined in an analogous manner as described for the above biomarker ELOVL 2 CpG 11, 12, 13, 14.

IV. FHL 2 CpG 13, 14, 15

In an analogous manner to the above biomarker FHL 2 CpG 11, 12, this biomarker is defined as the cumulative level of cytosine methylation at gene positions FHL 2 CpG 13, FHL 2 CpG 14, and FHL 2 CpG 15.

V. FHL 2 CpG 16, 17

In an analogous manner to the above biomarker FHL 2 CpG 11, 12, this biomarker is defined as the cumulative level of cytosine methylation at gene positions FHL 2 CpG 16, and FHL 2 CpG 17.

VI. Dehydroepiandrosterone sulfate (DHEAS)

This biomarker is defined as the concentration of DHEAS in plasma. DHEAS is an endogenous steroid hormone, produced in the adrenal glands, the gonads and the brain. It serves as a metabolic intermediate in the biosynthesis of androgens and estrogens. DHEAS is produced in the adrenal glands from dehydroepiandrosterone via the sulfotransferases SULT1A1 and SULT1E1.

VII. Log Ferritin

Ferritin is a ubiquitous intracellular protein that stores and releases iron in a controlled fashion.

VIII. S log p1/p6

This biomarker is defined as the common logarithm of the ratio between the concentration of NGA2F glycan in serum and the concentration of NA2F glycan in serum. NGA2F glycan is an agalactosylated core-α-1,6-fucosylated biantennary oligosaccharide, whereas NA2F glycan is a bigalactosylated core-α-1,6-fucosylated biantennary oligosaccharide.

IX. Plasma alpha-tocopherol

This biomarker is defined as the concentration of alpha-tocopherol in plasma. Alpha-tocopherol is a form of vitamin E.

X. S log p6 n glycan

This biomarker is defined as the common logarithm of the concentration of NA2F glycan in serum (see VIII, above).

XI. Alpha-2-macroglobulin

This biomarker is defined as the concentration of alpha-2-macroglobulin in serum. Alpha-2-macroglobulin is the largest major non-immunoglobulin protein in plasma. It acts as an anti-protease and inhibitor of both fibrinolysis and coagulation.

XII. Plasma lycopene

Lycopene is a carotene and carotenoid pigment and a phytochemical found in tomatoes. It is not an essential nutrient for humans, yet it has antioxidant activity and is transported in the blood by various lipoproteins. Lycopene accumulates in the liver, adrenal glands and testes.

XIII. Prostate specific antigen

This biomarker is defined as the concentration of prostate specific antigen (PSA) in serum. PSA a member of the kallikrein-related peptidase family and is secreted by the epithelial cells of the prostate gland. PSA is produced for the ejaculate, where it liquefies semen in the seminal coagulum and allows sperm to swim freely. PSA is present at low concentration in the serum of men with healthy prostates, but is often elevated in the presence of prostate cancer or other prostate disorders.

**SUPPLEMENTARY FIGURES**

Supplementary Figures 1-20 show the correlation of the respective biomarkers (y-axis) with chronological age (x-axis) in the male (S1-S10) and female (S11-S20) RASIG subgroup. The x-axis shows chronological age in years. The y-axis shows the respective biomarker values Z-score normalized. The number of subjects analysed, the function of the linear regression, and the parametric (rp) and non-parametric (rnp) correlation coefficients are shown on the top of each plot.

#

**Assessment of bioage score validity by genetic subgroup comparisons (GeHA Offspring vs. Spouses of GeHA Offspring) within the MARK-AGE study**

In order to perform an initial assessment of the validity for the biomarker sets, comparisons between GO and SGO subjects were performed. It is expected that age difference is lower in the GO compared to SGO and RASIG. In order to compare the different subgroups quantitatively, we selected a RASIG subgroup whose age distribution matches the one of the GO and SGO subgroups (Figure 3). Due to the high number of RASIG individuals, their mean value can be statistically separated from the one of the GO and SGO group with p-values below 1e-3 (Dunn’s multiple comparison test) but not between GO and SGO (p = 0.2272). However, since GO and SGO are recorded as couples, a paired test can be used for the comparison of the mean values between these subgroups. Here, the age difference appears to be normally distributed (D’Agostino and Pearson test, and Shapiro-Wilk test) but due to the small difference of means and the comparably small number of couples (100) the separation is less clear. A paired t-test of the hypothesis of equal means exhibits a p-value of 0.12 (with alternative hypothesis that age difference in SGO is greater than in GO).

**Supplementary Figure 21.** Comparison between MARK-AGE GO/SGO couples

**Supplementary Figure 22.** Chronological age versus biological age calculated by MARK-AGE score (red and blue) and Yang *et al*.[^106^](#_ENREF_106) biological age score based on clinical biomarkers (grey colour) in RASIG female (left panel) and RASIG male (right panel) cohort.

**Supplementary Figure 23.** Age difference of DS females (A) and males (B) compared to RASIG subgroup. Both, females and males were on average biologically older that RASIG (Statistical significance analysed by Mann-Whitney U test). Females Hodges-Lehmann median 5.258 and CI 2.126 to 8.489; Males Hodges-Lehmann median 3.901 and CI 0.8388 to 6.777


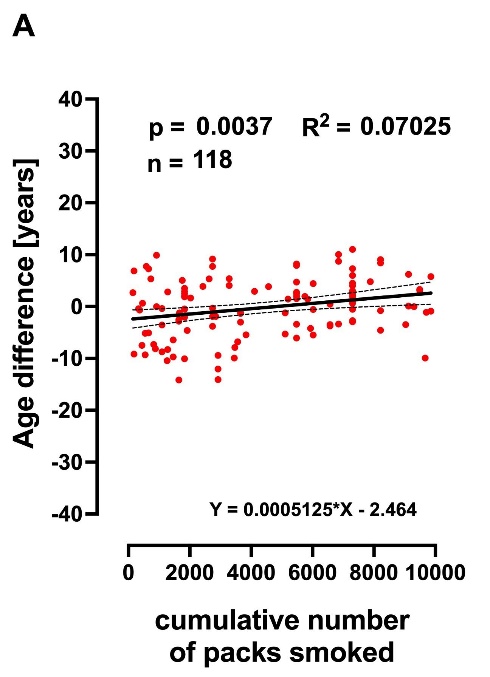

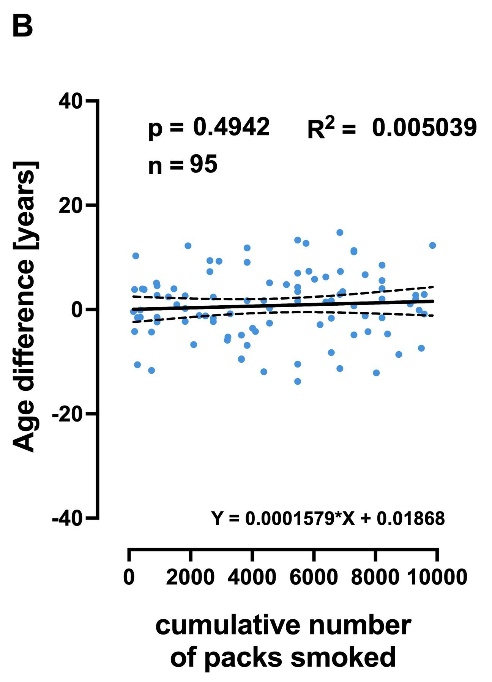


**Supplementary Figure 24.** Linear regression between cumulative number of packs of cigarettes smoked and age difference of female (A) and male (B) current smokers.

#

# Supplementary Figure 25. Effect of hormonal use in women aged 35-49 (A) and women older than 50 years (B). For women between 35 and 49 years, there is no significant age difference as a function of hormone use, whereas 50-70 years old women taking HRT were biologically younger. (A) Hodges-Lehmann median 0.7042 and CI -0.8536 to 2.301; (B) Hodges-Lehmann median -2.178 and CI -3.139 to -1.192

#
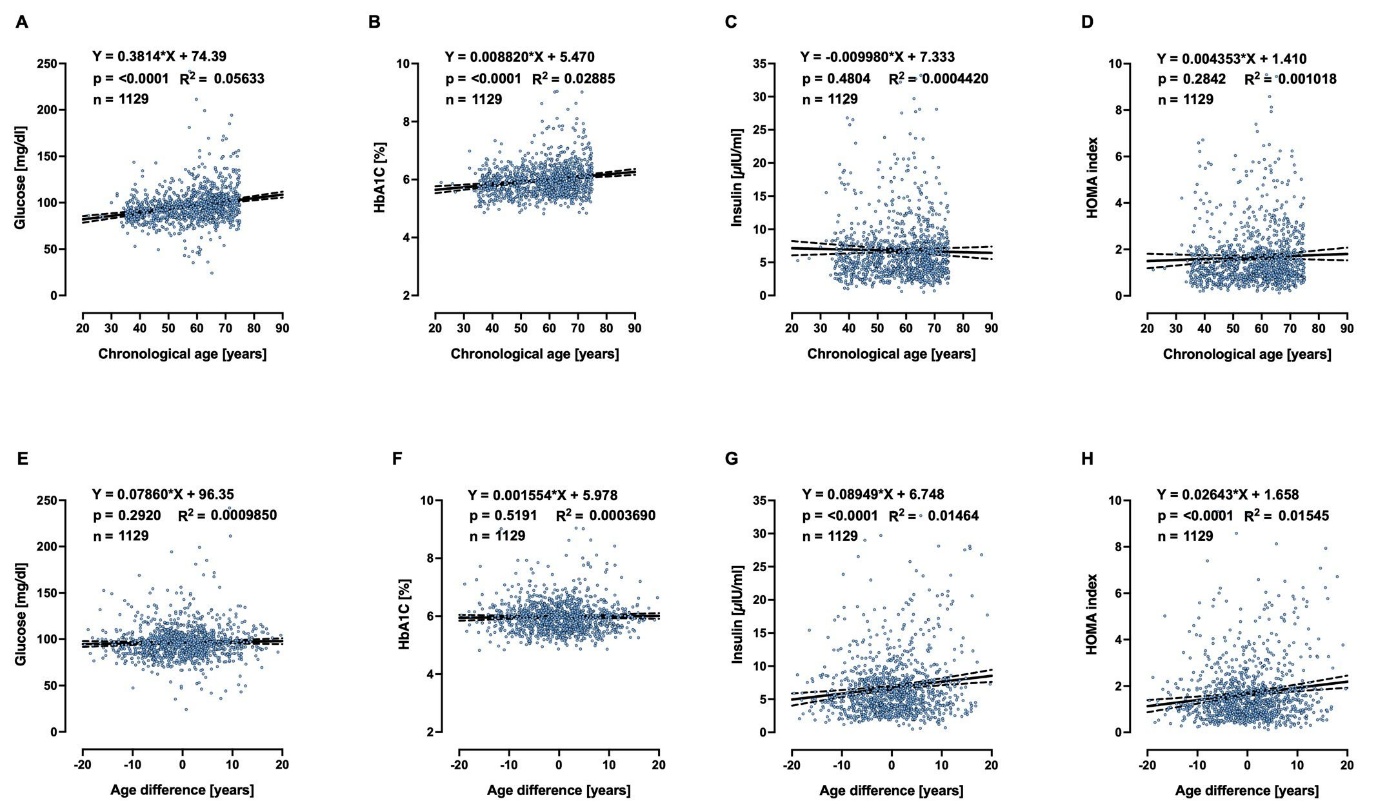


# Supplementary Figure 26. Linear regressions of chronological age and age difference on fasting glucose, HbA1c, fasting insulin and HOMA index in males. A and E: Glucose; B and F: HbA1C; C and G: Insulin; D and H: HOMA index.

#

#
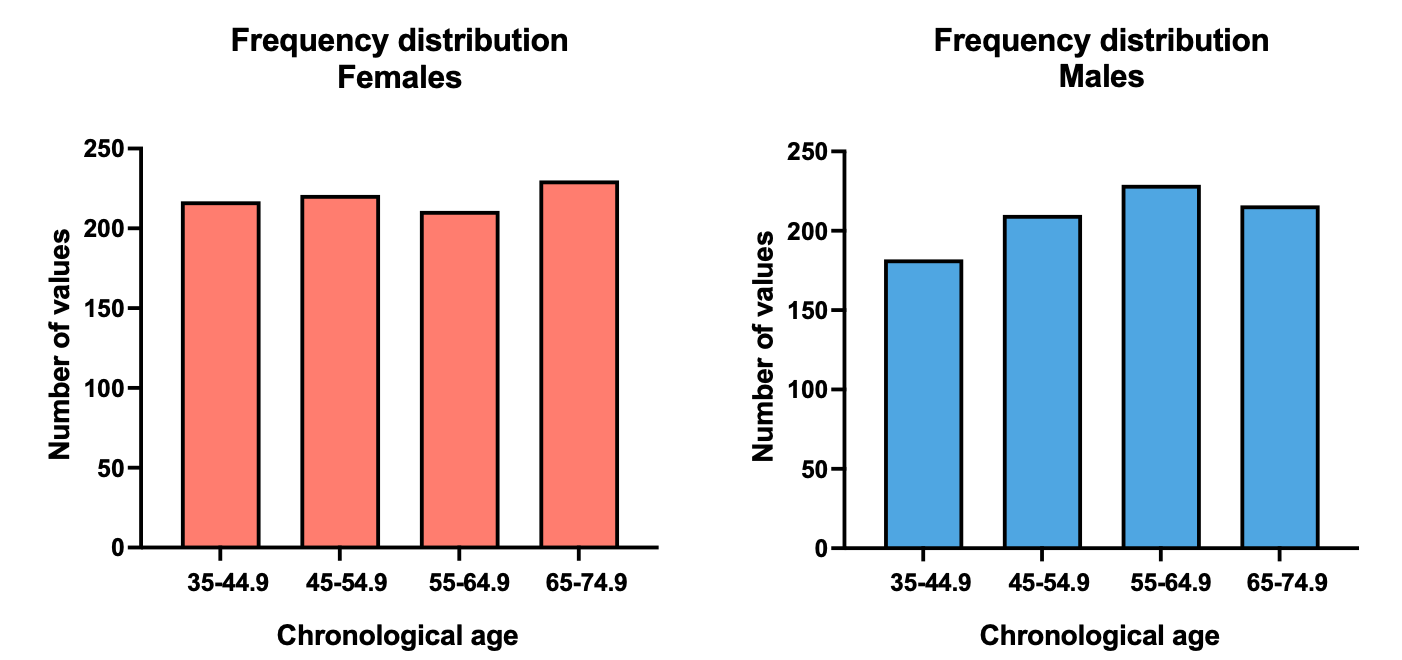


# Supplementary Figure 27: Frequency distribution in MARK-AGE female population (red) and MARK-AGE male population (blue). Each 10-year group is represented with similar number of subjects.

# SUPPLEMENTARY TABLES

## **Supplementary Table 1**: Values for $\bar{x}_{i}$, σi, bi, c and r^2^ for females

| Parameter *i* | $\bar{x}_{i}$ | *σ_i_* | *b* | *c* | *r^2^* |
| --- | --- | --- | --- | --- | --- |
| ELOVL2 CpG 11.12.13.14 | 0.6259 | 0.0704 | 3.0278 |  |  |
| ELOVL2 CpG 15.16.17 | 0.5658 | 0.0642 | 2.8692 |  |  |
| FHL2 CpG 11.12 | 0.2042 | 0.0458 | -0.8935 |  |  |
| FHL2 CpG 13.14.15 | 0.3310 | 0.0539 | 1.6160 |  |  |
| FHL2 CpG 16.17 | 0.5045 | 0.0931 | 1.2488 |  |  |
| Dehydroepiandrosterone  sulfate | 2.800 | 1.7671 | -1.3506 |  |  |
| S p6 n glycan | 19.3033 | 3.7579 | -1.1485 |  |  |
| Log ferritin | 1.5606 | 0.431 | 1.5740 |  |  |
| S log p1/p6 | -0.3467 | 0.2117 | 1.7216 |  |  |
| Plasma $\alpha$-tocopherol | 29.0112 | 7.7987 | 0.7425 |  |  |
|  |  |  |  | 55.185 | 0.877 |

## **Supplementary Table 2**: Values for xi, σi, bi, c and r^2^ for males

| Parameter *i* | *x_i_* | *σ_i_* | *b* | *c* | *r^2^* |
| --- | --- | --- | --- | --- | --- |
| ELOVL2 CpG 11.12.13.14 | 0.6250 | 0.0721 | 3.1171 |  |  |
| ELOVL2 CpG 15.16.17 | 0.5636 | 0.0633 | 2.7062 |  |  |
| FHL2 CpG 11.12 | 0.2000 | 0.0415 | -1.4602 |  |  |
| FHL2 CpG 13.14.15 | 0.3279 | 0.0541 | 2.5376 |  |  |
| FHL2 CpG 16.17 | 0.5023 | 0.0945 | 1.6110 |  |  |
| Dehydroepiandrosterone  sulfate | 4.5465 | 2.8269 | -2.3933 |  |  |
| S p6 n glycan | 19.3520 | 2.8775 | -1.0011 |  |  |
| $\alpha$2-macroglobulin | 117.348 | 34.0189 | 0.8075 |  |  |
| Lycopene | 0.7627 | 0.4284 | -1.3033 |  |  |
| Log prostate specific antigen | 0.0013 | 0.4168 | 1.0869 |  |  |
|  |  |  |  | 55.686 | 0.842 |

**Supplementary Table 3**: Age difference of each DS female subject

| Sex | Chronological age | Bioage | AgeDiff |
| --- | --- | --- | --- |
| F | 63 | 70.55937 | 7.59369 |
| F | 39 | 48.6911 | 9.691101 |
| F | 52 | 54.8319 | 2.831896 |
| F | 30 | 37.06926 | 7.069256 |
| F | 55 | 52.09884 | -2.90116 |
| F | 30 | 43.24905 | 13.24905 |
| F | 37 | 38.59908 | 1.599083 |
| F | 43 | 52.61808 | 9.618084 |
| F | 34 | 29.89725 | -4.10275 |
| F | 50 | 52.34014 | 2.340142 |
| F | 39 | 49.33255 | 10.33255 |
| F | 59 | 57.42951 | -1.57049 |
| F | 26 | 38.54562 | 12.54562 |
| F | 19 | 28.7214 | 9.721395 |
| F | 19 | 22.68933 | 3.689331 |
| F | 68 | 61.58239 | -6.41761 |
| F | 66 | 59.7651 | -6.2349 |
| F | 26 | 33.72689 | 7.726885 |
| F | 45 | 64.39764 | 19.39764 |

**Supplementary Table 4**: Age difference of each DS male subject

| Sex | Age | Bioage | AgeDiff |
| --- | --- | --- | --- |
| M | 63 | 72.06841 | 9.068415 |
| M | 56 | 59.03356 | 3.033562 |
| M | 39 | 39.75522 | 0.755218 |
| M | 36 | 43.3227 | 7.322697 |
| M | 39 | 53.68526 | 14.68526 |
| M | 39 | 47.2325 | 8.232502 |
| M | 45 | 50.76498 | 5.764984 |
| M | 49 | 63.82157 | 14.82157 |
| M | 30 | 38.26414 | 8.264142 |
| M | 36 | 38.14167 | 2.14167 |
| M | 34 | 32.74616 | -1.25384 |
| M | 45 | 50.72509 | 5.725088 |
| M | 32 | 37.79062 | 5.790621 |
| M | 32 | 29.11718 | -2.88282 |
| M | 27 | 26.3849 | -0.6151 |
| M | 26 | 23.43739 | -2.56261 |
| M | 58 | 58.03712 | 0.037117 |
| M | 22 | 13.91614 | -8.08386 |
